# Metabolite and Transcriptome Profiling Analysis Provides New Insights into the Distinctive Effects of Exogenous Melatonin on Flavonoids Biosynthesis in *Rosa rugosa*

**DOI:** 10.3390/ijms25179248

**Published:** 2024-08-26

**Authors:** Yong Xu, Ruotong Wang, Yuanxiao Ma, Meng Li, Mengjuan Bai, Guo Wei, Jianwen Wang, Liguo Feng

**Affiliations:** College of Horticulture and Landscape Architecture, Yangzhou University, Yangzhou 225009, China; yongxu@yzu.edu.cn (Y.X.); wangruotong_0809@163.com (R.W.); myx000127@163.com (Y.M.); mz120231532@stu.yzu.edu.cn (M.L.); baimengjuan@yzu.edu.cn (M.B.); jwwang@yzu.edu.cn (J.W.)

**Keywords:** melatonin, flavonoids, metabolomic and transcriptomic analyses, virus-induced gene silencing

## Abstract

Although the petals of *Rosa rugosa* are rich in flavonoids and their bioactivity has a significant impact on human health, the flavonoid content decreases during flower development. In this study, *R. rugosa* ‘Feng hua’ was used to investigate the effects of the melatonin foliar spray on enhancing the quality of rose by focusing on major flavonoids. The results showed that the contents of total flavonoids in rose petals at the full bloom stage induced by melatonin obeyed a bell-shaped curve, with a maximum at 0.3 mM, indicating the concentration-dependent up-regulation of flavonoid biosynthesis. In the treatment with 0.3 mM melatonin, metabolomic analyses showed that the concentrations of ten main flavonoids were identified to be increased by melatonin induction, with high levels and increases observed in three flavonols and two anthocyanins. KEGG enrichment of transcriptomic analysis revealed a remarkable enrichment of DEGs in flavonoid and flavonol biosynthesis, such as *Rr4CL*, *RrF3H*, and *RrANS*. Furthermore, functional validation using virus-induced gene silencing technology demonstrated that *Rr4CL3* is the crucial gene regulating flavonoid biosynthesis in response to the stimulant of melatonin. This study provides insights into the exogenous melatonin regulation mechanism of biosynthesis of flavonoids, thereby offering potential industrial applications.

## 1. Introduction

*Rosa rugosa*, a deciduous shrub belonging to the Rosaceae family, has significant ornamental and economic value. It is classified as a plant with medicinal and edible uses, which contains diverse functional compounds essential for human metabolism, such as volatile organic compounds, flavonoids, polyphenols, and polysaccharides. These compounds enable roses to serve as essential raw materials in the production of various products, including floral teas, confections, jams, biscuits, and beverages within the food industry [1,2]. Experimental evidence has demonstrated that bioactive components in roses, particularly flavonoids, anthocyanins, and essential oils, possess antioxidant, antimicrobial, anti-inflammatory, antidiabetic, and anticancer properties [3]. The total flavonoid contents of *R. rugosa* vary from 1.37 mg RE/g to 7.21 mg RE/g (milligrams of rutin equivalents per gram of dry weight) [4]. Flavonoids, which play essential roles in many biological processes and responses to environmental factors in plants, positively affect human health mainly because of their strong bioactivities [5,6]. However, the flavonoid contents decrease during flower development in *R. rugosa* [7].

In recent years, secondary metabolism has gained increasing interest because of its commercial potential. Various strategies, including biotic (proteins, carbohydrates, plant growth promoting rhizobacteria, fungus, hormones), abiotic elicitation (heavy metals, low and high temperature, light, salt, drought), and biostimulants (humic substances (HS), protein hydrolysates, seaweed extracts, and microorganisms), have been explored to enhance secondary metabolite production in plants [8,9]. Melatonin is an endogenous molecule highly conserved in eukaryotes and structurally similar to auxins. It functions as a novel growth regulator and biostimulant. It is well known that melatonin improves plant resistance to various biotic and abiotic stresses by effectively inhibiting oxidative stress by enhancing plant antioxidant enzyme activity and antioxidant gene expression through mostly unknown nuclear receptors [10,11]. It also exerts its effects through potent activation of MAPK cascades, thereby mediating numerous critical growth, developmental, and metabolic processes in plants [12]. Recent studies have highlighted the immense potential of melatonin in regulating various aspects of horticultural plants, serving as a key regulator of gene expression, developmental control, and stress mitigation [13].

Melatonin also plays roles in seed germination, plant growth, and flowering induction and exhibits concentration-dependent effects [14,15]. For example, stevia seedlings germinated showed significantly inhibited growth of aerial parts at 500 μM melatonin, while it appears to be toxic to its growth at high concentrations [16]. The flower number was decreased and the flowering time was delayed for apple trees when the concentration of melatonin was too high [14]. Melatonin treatments enhance fruit ripening and improve the quality of horticultural plants by increasing the levels of beneficial substances, such as sucrose, natural antioxidants, phenolic compounds, aroma constituents, polyphenols, and soluble solids [17]. For instance, melatonin application increases soluble sugars, particularly sucrose and sorbitol, during pear fruit ripening [18]. It also enhances the essential oil content in medicinal lemon verbena leaves [19]. The melatonin-induced expression of *VvMYB14* promotes ethylene production, thereby accelerating grape fruit ripening and altering the accumulation of metabolic products; this phenomenon increases total phenols, flavonoids, and proanthocyanidins in grape skins, including resveratrol and its derivatives [20]. Moreover, melatonin improves fruit quality postharvest, thereby delaying senescence and extending shelf life [21]. Studies have also shown that melatonin increases the accumulation of phenolic and flavonoid compounds in fruits post-application [22,23]. For example, melatonin application in strawberries reduces fruit decay and increases the accumulation of total phenols and anthocyanins [24].

In addition to directly enhancing plant antioxidant capacity, melatonin also regulates the abiotic stress tolerance of plants through regulation of photosynthetic system, primary metabolism, and secondary metabolism (such as total flavonoid, phenolic, and essential oil contents) under abiotic stress conditions, including water, temperature, saline, and heavy metal stress [25]. For instance, seeds primed with 10 μM of melatonin solution showed improved tolerance of basil (*Ocimum basilicum*) to salinity stress, by increasing the levels of total phenolics and flavonoids [26]. However, under normal, non-stressful conditions, melatonin tends to inhibit anthocyanin biosynthesis in Arabidopsis [27]. Additionally, melatonin coordinates with plant hormones to regulate plant growth and development; in this process, auxins, gibberellins, abscisic acid, ethylene, salicylic acid, and polyamines are involved in melatonin signaling pathways, thereby forming a complex signaling network that controls horticultural crop growth and development [21]. As an excellent biostimulant, exogenous melatonin has significant effects on promoting seed germination, and plant growth, enhancing plant adaptation to abiotic stress conditions, as well as improving crop yield and quality.

Melatonin has a recognized role as a growth stimulant that regulates various biochemical processes in plants, particularly in increasing beneficial secondary metabolites, such as phenolics, flavonoids, anthocyanins, and plant essential oils. However, research on its molecular mechanisms in regulating secondary metabolites in roses is limited. This study applies different concentrations of exogenous melatonin via foliar spray on *R. rugosa* to identify the optimal concentration for enhancing rose quality by focusing on major flavonoids. This study aims to elucidate the main components and related genes affected by melatonin in rose flavonoids through integrated metabolomic and transcriptomic analyses. Furthermore, the functional validation of key genes responsive to melatonin regulation in roses is conducted using virus-induced gene silencing (VIGS) technology, thereby preliminarily exploring the potential mechanisms by which melatonin enhances rose quality. This research lays the foundation for understanding the role of exogenous melatonin in regulating secondary metabolites in roses and provides insights into the molecular basis of flavonoids in rose flowers, offering a reference for achieving efficient and high-quality cultivation of *R. rugosa* using melatonin.

## 2. Results

### 2.1. Effect of Exogenous Melatonin on the Main Quality Indices of Rose Petals

**Figure 1:**
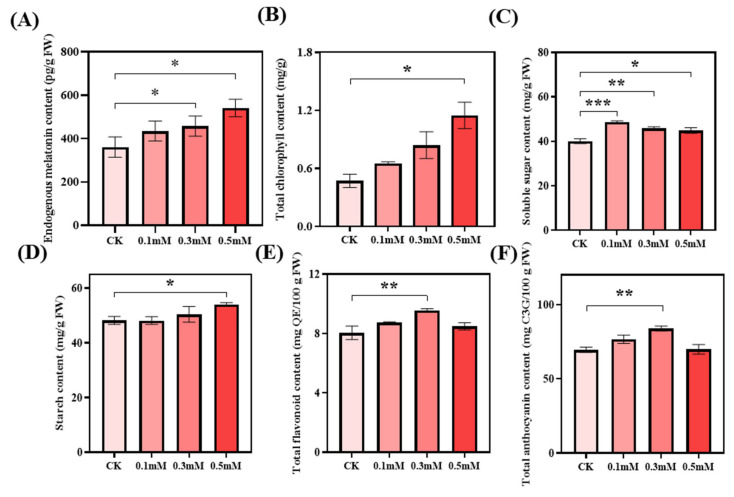
The content of the main quality indexes of *R. rugosa* affected by exogenous melatonin: (**A**) Endogenous melatonin. (**B**) Total chlorophyll. (**C**) Soluble sugar. (**D**) Starch. (**E**) Total flavonoid. (**F**) Total anthocyanin. Note: CK means the control; Asterisks denote statistically significant differences (* *p* < 0.05; ** *p* < 0.01; *** *p* < 0.001).

As the melatonin solution was mainly sprayed on leaves (Section 4.1), and the amount of melatonin absorbed by the tightly closed bud of the flower was rare, melatonin was synthesized by itself at the flowering stage. The experimental results demonstrate that the endogenous melatonin content in rose petals increases with the application of different exogenous melatonin concentrations. The maximum melatonin content is observed at the 0.5 mM level of exogenous melatonin concentration. Compared with the control (zero melatonin), the groups treated with an exogenous melatonin concentration of 0.3 mM and 0.5 mM show approximately 26.8% and 50.1% significant increases in melatonin content, respectively (Figure 1A). The total chlorophyll content in rose leaves increases with increasing exogenous melatonin. The chlorophyll content at 0.5 mM level is the highest, approximately 143.0% higher than that of the control, with a significant difference (Figure 1B). The soluble sugar content in rose petals shows a trend of initially increasing and then decreasing with increasing exogenous melatonin concentrations. The peak of soluble sugar content is reached with the melatonin concentration of 0.1 mM (Figure 1C). Treatments with 0.1, 0.3, and 0.5 mM melatonin concentrations result in approximately 21.4%, 14.5%, and 12.1% increases in soluble sugar content, respectively. These values show significant differences from the soluble sugar content of the control. The starch content in rose petals gradually increases with the exogenous melatonin concentration (Figure 1D). Its maximum is reached at the 0.5 mM concentration level. Compared with the control, the group treated with exogenous melatonin at a concentration of 0.5 mM shows a significant increase in starch content by approximately 12.1% (Figure 1C). The total flavonoid and total anthocyanin contents in rose petals initially increase and then decrease with increasing concentrations of melatonin (Figure 1E,F). They both peak at the 0.3 mM level of exogenous melatonin concentration. Compared with the control, the group treated with exogenous melatonin concentration of 0.3 mM shows approximately 18.9% and 20.7% increases in total flavonoid and total anthocyanin contents, respectively, with significant differences observed.

### 2.2. Effect of Exogenous Melatonin on Flavonoid Composition and Content in Rose Petals

**Figure 2:**
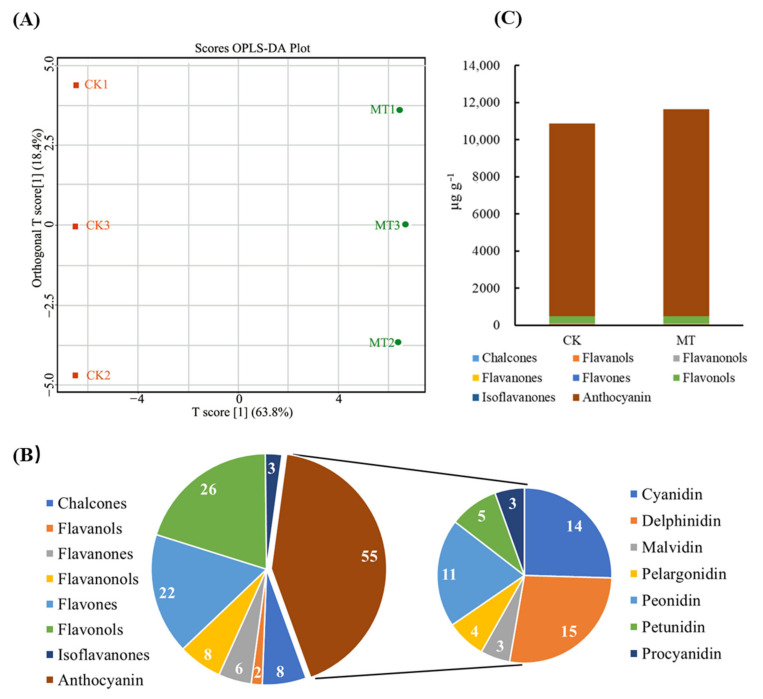
Effect of exogenous melatonin on flavonoid composition and content in rose petals: (**A**) The OPLS-DA model analysis. (**B**) The number of different kinds of flavonoid species and the composition of anthocyanin. (**C**) The content of different kinds of flavonoids with and without treated by melatonin in petals.

A total of 130 flavonoids were detected based on the metabolomic analyses. The OPLS-DA model is reliable with an R2Y value of 0.998 and a Q2 value of 0.99, and it indicates that the samples show good repeatability within each group (18.4%) and significant differences between the two groups (63.8%) (Figure 2A). In terms of the number of species, the petals of rose in full bloom contain eight kinds of flavonoids, among which anthocyanin, flavonols, and flavones have the most components, with 55, 26, and 22 species, respectively. Anthocyanins can be divided into seven types, among which delphinidin, cyanidin, and peonidin have the most components, with 15, 14, and 11 species, respectively (Figure 2B). The total content of flavonoids increases from 11,432.3 ± 339.7 μg g^−1^ to 12,169.4 ± 641.4 μg g^−1^ (Figure 2C). Anthocyanin has the highest content, accounting for ~95% of flavonoid content. After melatonin treatment, its content increases from 10,385.4 ± 546.3 μg g^−1^ to 11,139.4 ± 805.1 μg g^−1^, an increase of ~7.3%. Anthocyanin is followed by flavonols, accounting for ~3% of flavonoid content. The content of flavonols increases from 346.76 ± 40.6 μg g^−1^ to 400.6 ± 3.6 μg g^−1^, an increase of ~15.5%. In addition, the highest content of anthocyanin is peonidin, which accounts for ~64% of the total anthocyanins. The content of peonidin increases from 6640.8 ± 451.9 μg g^−1^ to 7311.9 ± 474.2 μg g^−1^, an increase of ~10.1%. Cyanidin follows, accounting for ~33% of the total anthocyanins. Its content increases from 3449.8 ± 126.2 μg g^−1^ to 3596.8 ± 311.3 μg g^−1^, an increase of ~4.3%.

### 2.3. Effect of Exogenous Melatonin on the Contents of the Main Components of Flavonoids in Rose Petals

**Figure 3:**
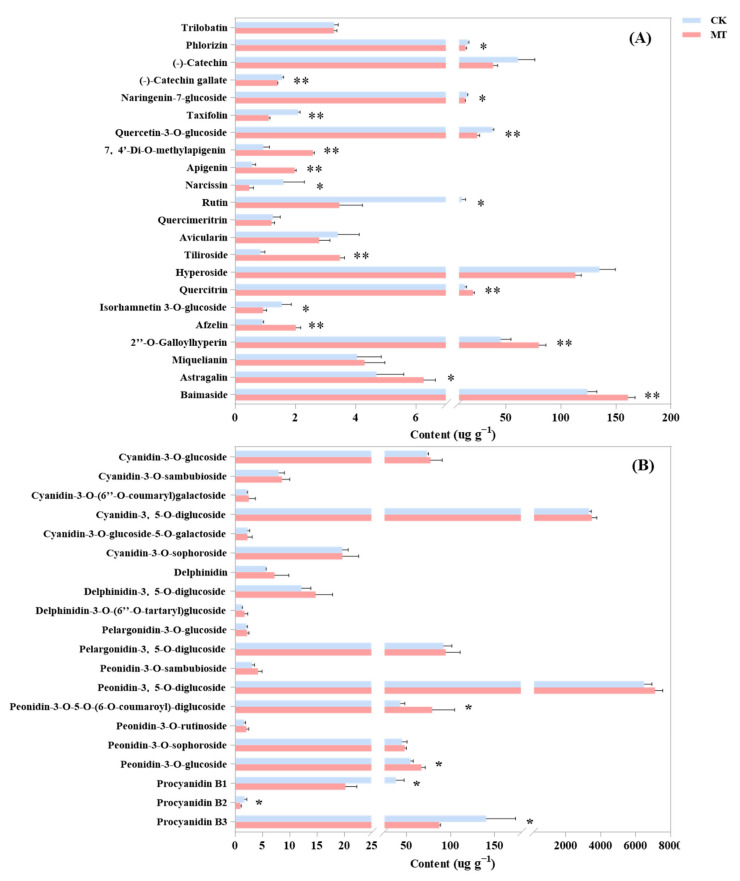
Effects of exogenous melatonin on the main flavonoid components with a content greater than 1 μg g^−1^ in R. rugosa: (**A**) The content of each component of flavonoids except for anthocyanin. (**B**) The content of each component of anthocyanin. Asterisks denote statistically significant differences (* *p* < 0.05; ** *p* < 0.01).

The VIP value and the FC value of the OPLS-DA model were combined to screen the differential metabolites. A total of 21 flavonoids with significant differences, including 1 chalcone, 1 flavanol, 1 flavanone, 2 flavanonols, 3 flavones, 8 flavonols, and 5 anthocyanins (including 2 peonidins and 3 procyanidins), were screened with the content greater than 1 μg g^−1^ (Figure 3A,B). Ten species are significantly upregulated, among which quercitrin, 2″-o-galloylhyperin, and baimaside in flavonols are high, and the increase rate is large (Figure 3A). Melatonin promotes the average increase of 7.3 μg g^−1^ (the increase rate is 57.8%), 34.57 μg g^−1^ (76.4%), and 36.77 μg g^−1^ (29.6%) for the three components. In anthocyanins, the contents of peonidin-3-o-5-o-(6-o-coumaroyl)-diglucoside and peonidin-3-o-glucoside are significantly high, with melatonin increasing them by 36.0 μg g^−1^ (83.5%) and 12.2 μg g^−1^ (22.3%), respectively (Figure 3B).

### 2.4. Transcriptomic Analysis of Exogenous Melatonin Regulation of Rose Quality

**Figure 4:**
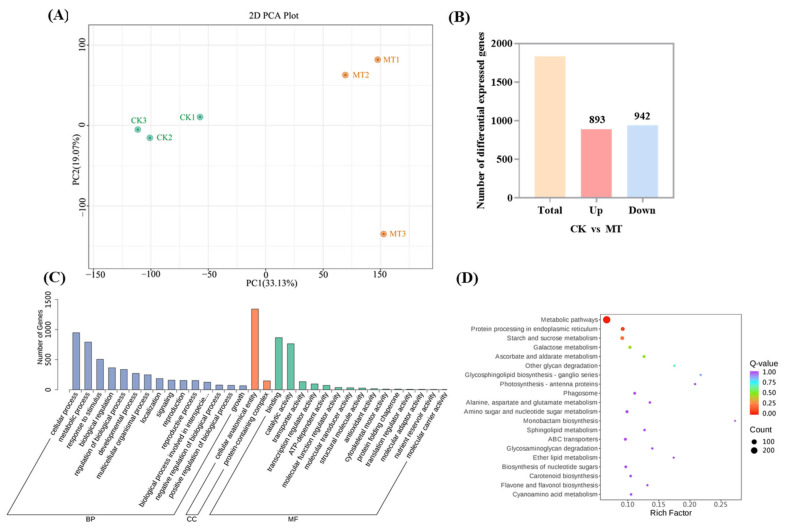
Analysis of Differential Expressed Genes (DEGs): (**A**) The principal component analysis. (**B**) Counts of upregulated and downregulated genes. (**C**) Gene Ontology Analysis of DEGs. (**D**) Kyoto Encyclopedia of Genes and Genomes pathway enrichment analysis of DEGs.

The RNA sequencing results indicate that all samples achieve more than 7 Gb of clean base, with Q20 values exceeding 97.37% and Q30 values over 92.49%. This finding indicates a high sequencing quality suitable for transcriptomic profiling. The clean reads are aligned to the reference genome of the wild rose. Thus, the average alignment rates are 89.70% for clean reads aligning to genes and 84.39% for clean reads aligning uniquely to gene locations (Table S2). The distribution of FPKM values across samples depicted in the box plot (Figure S1) shows no significant deviation in overall expression abundance, thereby facilitating subsequent analyses. The principal component analysis reveals significant variations in the gene expression among different treatments. The first two principal components account for 33.13% (PC1) and 19.07% (PC2) (Figure 4A).

A total of 1835 DEGs are identified through differential gene expression analysis, with 892 genes upregulated and 943 genes downregulated in response to melatonin induction (Figure 4B). GO enrichment analysis of the identified DEGs reveals significant enrichment primarily in biological processes, such as cellular and metabolic processes, followed by response to stimulus (Figure 4C). Within cellular components, the enrichment is notable in the cellular anatomical entity, whereas molecular functions are enriched in binding and catalytic activities. The KEGG pathway enrichment analysis (Figure 4D) highlights that DEGs are significantly enriched in metabolic pathways (44.43%). The key pathways include starch and sucrose metabolism, galactose metabolism, and flavone and flavonol biosynthesis, indicating their involvement in the regulatory processes influenced by exogenous melatonin.

### 2.5. Expression Pattern Analysis of DEGs Involved in Flavonoid Compound Biosynthesis Pathways

**Figure 5:**
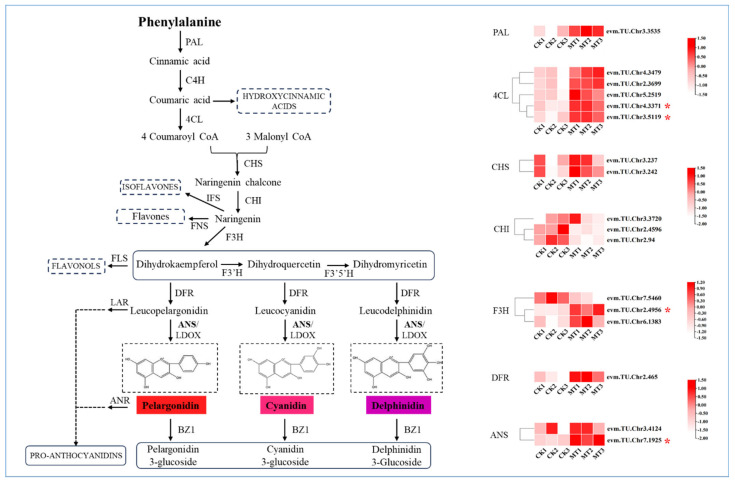
Schematic Diagram of Metabolic Pathways of Flavonoids. Transcriptome data were utilized to analyze the expression levels of structural genes. Different colored boxes indicate changes in gene expression levels. The enzymes are abbreviated as follows: PAL, phenylalanine ammonia-lyase; C4H, cinnamate-4-hydroxylase; 4CL, 4-coumarate: coenzyme A ligase; CHS, chalcone synthase; CHI, chalcone isomerase; IFS, isoflavone synthase; FNS, flavone synthase; F3H, flavanone-3-hydroxylase; F3′H, flavonoid 3′-hydroxylase; F3′5 ′H, flavonoid 3′,5′-hydroxylase; FLS, flavonol synthase; LAR, leucoanthocyanidin reductase; DFR, dihydroflavonol 4-reductase; ANS, anthocyanin synthase; ANR, anthocyanidin reductase; BZ1, bronze 1. Asterisks denote statistically significant differences (* *p* < 0.05).

The expression levels of genes involved in the flavonoid biosynthesis pathway were analyzed to investigate further the effects of exogenous melatonin treatment at the molecular level. Several DEGs were annotated (Figure 5). Two 4-coumarate-CoA ligases (*4CL*) and one flavanone 3-hydroxylase (*F3H*) are significantly upregulated upon melatonin induction. Phenylalanine ammonia-lyase (PAL), a key enzyme upstream in this synthesis pathway, is also influenced by melatonin. Downstream in the anthocyanin biosynthesis branch pathway of flavonoid biosynthesis, dihydroflavonol reductase (*DFR*) and anthocyanidin synthase (*ANS*) are induced by exogenous melatonin, with *ANS* showing significant upregulation as a crucial enzyme at the terminal step of anthocyanin synthesis.

**Figure 6:**
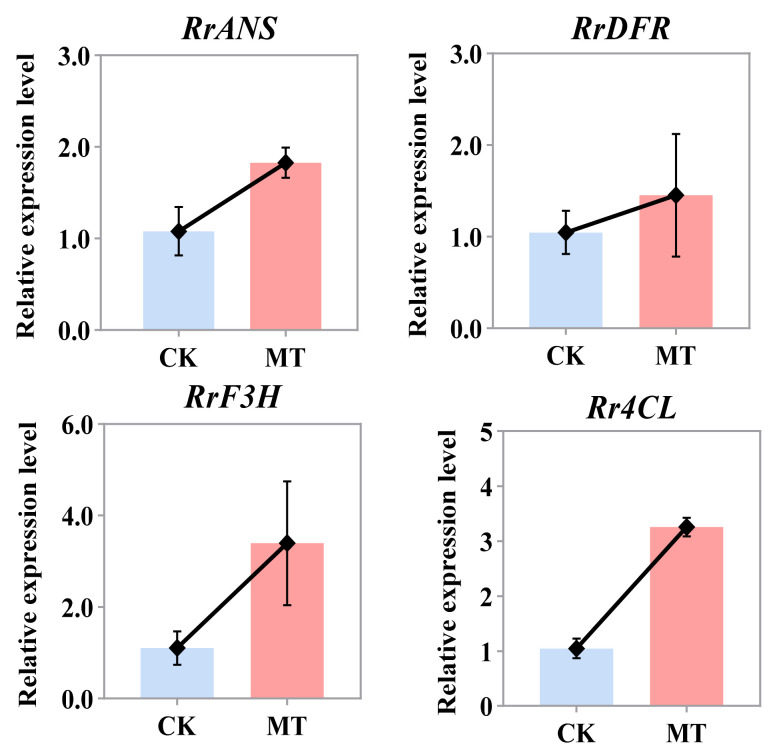
Relative expression levels of 4 selected candidate structural genes involved in the flavonoid metabolic pathway determined by qRT-PCR.

We employed qRT-PCR technology to verify the expression patterns of the selected genes (including, *Rr4CL*, *RrF3H*, *RrDFR*, and *RrANS*) and validate the accuracy of the RNA-seq results (Figure 6). The results confirm that the key enzymes involved are upregulated during the peak flowering period under exogenous melatonin induction. This finding is consistent with the trends observed in the RNA-seq data, thereby confirming the reliability and accuracy of the transcriptomic sequencing results.

### 2.6. Functional Validation of Candidate Gene Rr4CL3 in Regulating Flavonoid Compound Content in Rose Petals

**Figure 7:**
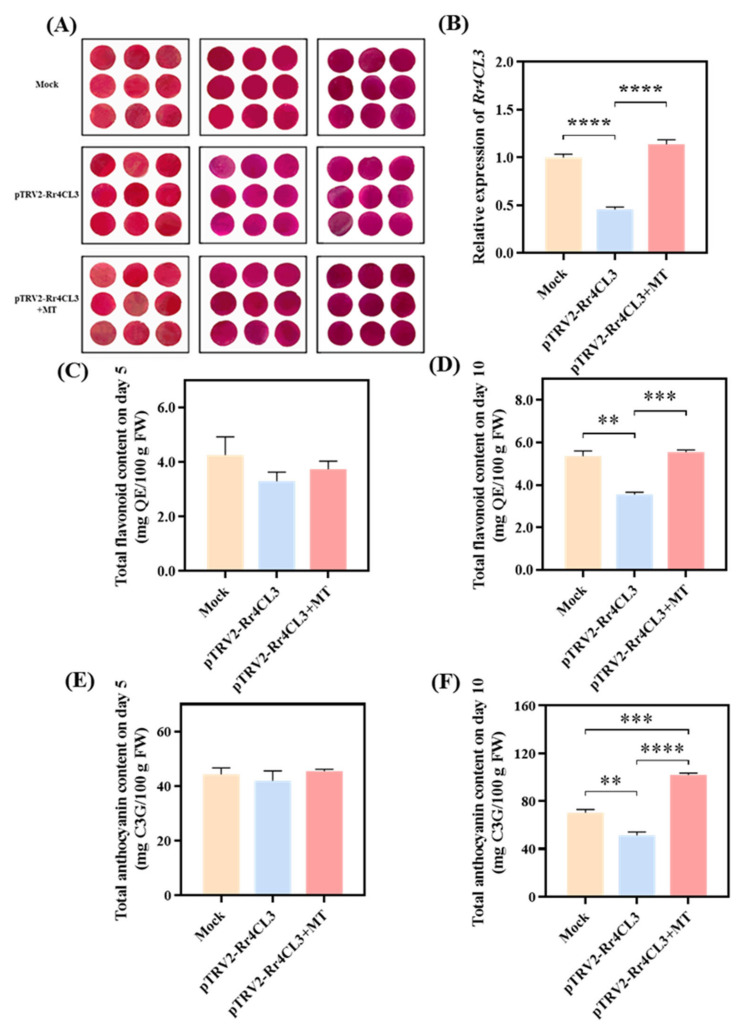
Virus-induced silencing of *Rr4CL3* in rose petals: (**A**) The phenotype of petals on different days. (**B**) Change in *Rr4CL3* gene expression level. (**C**) Total flavonoid content on day 5. (**D**) Total flavonoid content on day 10. (**E**) Total anthocyanin content on day 5. (**F**) Total anthocyanin content on day 10. Asterisks denote statistically significant differences (** *p* < 0.01; *** *p* < 0.001 **** *p* < 0.0001).

*Rr4CL* (Gene ID: evm.model.Chr3.5119) was characterized based on its nucleotide sequence. The ORF of *Rr4CL3* is 1767 bp, encoding 589 amino acids. Compared with similar genes using NCBI BLAST, the *Rr4CL* gene is similar to *At4CL3* in Arabidopsis Thaliana, which regulates flavonoids, so we named this gene *Rr4CL3* (Figure S2). It was transiently silenced using Agrobacterium-mediated transformation in R. chinensis Jacq. ‘Crimson Glory’ petals (Figure 7A). As *Rr4CL3* is involved in regulating the content of flavonoid compounds in rose petals, it potentially promotes the synthesis of flavonoids by increasing *Rr4CL3* expression levels under the stimulation of melatonin. The role of *Rr4CL3* was validated further by VIGS.

Compared with the control group, *R. rugosa* ‘Black Baccara’ petals that underwent transient silencing of *Rr4CL3* show a significant decrease in gene expression by approximately 54%. This finding confirms successful gene silencing (Figure 7B). Furthermore, the relative expression level of *Rr4CL3* in silenced petals after melatonin treatment increases by approximately 147.8%, which is significantly different from that in silenced petals without melatonin treatment (Figure 7B). The petals with silenced *Rr4CL3* showed light coloration from day 5, which slightly darkened by day 10 (Figure 7A). However, they consistently remain lighter than the control at the same time points. Compared with the control after 5 days, the silenced petals show a decrease in the contents of flavonoid compounds and anthocyanins by approximately 22.35% and 5.42%, respectively (Figure 7C,E). Moreover, the contents of flavonoid compounds and anthocyanins in silenced petals increased by day 10 (Figure 7D,F) compared with those in day 5. However, they remained lower than the contents in the control, with reductions of approximately 33.58% and 26.68% for flavonoids and anthocyanins, respectively. This finding shows silenced petals’ significant differences from the control. These findings indicate that *Rr4CL3* plays an important role in the accumulation of flavonoids and anthocyanins in roses.

After 5 days, the color of the silenced petals treated with melatonin deepened compared to those silenced with *Rr4CL3*. Correspondingly, the contents of flavonoids and anthocyanins also increased, by approximately 13.33% and 8.43%, respectively (Figure 7D,F). After 10 days, the color difference between the silenced petals treated with melatonin and those without melatonin treatment became more pronounced. The flavonoid content in the silenced petals after 10 days of melatonin treatment increased by approximately 56.06% (Figure 7D), approaching a level similar to the control, while the anthocyanin content increased by approximately 98.04%, which was about 45.20% higher than the control (Figure 7F).

## 3. Discussion

Compared with the control, the content of flavonoid compounds was improved in rose petals with 0.3 mM melatonin treated by upregulating genes of flavonoid biosynthesis pathways, which should be attributed to a significant increase in endogenous melatonin content [17]. It has been studied that endogenous melatonin could be induced by exogenous melatonin in plants [28,29,30]. In addition, the transport-based inclusion of melatonin from the outside by transmembrane receptors, such as MTR1/CAND2, to freely travel through membranes could also be part of the increase in endogenous melatonin [31,32]. However, as its role in plants is complex, melatonin transport is still poorly understood, thereby further studies are needed to explore the possibility of novel signaling mechanisms in how the exogenous melatonin treatment enhances the content of endogenous melatonin [33]. In this study, melatonin treatments at different concentrations consistently increased chlorophyll content in rose leaves; this is because exogenous melatonin promotes the accumulation of photosynthetic pigments, thereby enhancing photosynthetic efficiency [14,34]. Exogenous melatonin likely enhances photosynthetic pigment accumulation, strengthens photosynthetic capacity, and promotes the accumulation of primary metabolites, such as soluble sugars and starch in rose plants, thereby indirectly facilitating the accumulation of polyphenolic compounds [35]. One of the reasons for the accumulation of polyphenolic substances induced by exogenous melatonin in roses may be the high levels of carbohydrates, such as glucose and sucrose, which lead to increased levels of polyphenolic compounds, such as anthocyanins [36]. Notably, further research on the regulation pathway of whether the exogenous melatonin is able to promote the biosynthesis of flavonoids, through modulating the hormone network (including the endogenous melatonin), should be based on the accurate measurement of these hormones with LC/MS-MS [28].

This study demonstrates that flavonoids and flavonols are key flavonoid compounds significantly affected by melatonin signaling, possibly because melatonin activates key enzymes in the flavonoid and flavonol biosynthesis pathways. Transcriptomic analysis indicates that melatonin may enhance the accumulation of flavonoid compounds in rose petals by activating the expression levels of genes in the flavonoid biosynthesis pathway. As the first enzyme in the phenylpropane metabolic pathway, PAL serves as a direct or indirect precursor for many secondary metabolites. Functioning as a pivotal hub in phenylpropane and flavonoid metabolism, 4CL plays important roles in flavonoid biosynthesis [6,37]. Enhanced enzyme activity induced by melatonin can lead to the production and accumulation of additional upstream metabolites, such as naringenin chalcone and naringenin in roses, which are significant differential metabolites consistent with the expression patterns of upstream key genes *RrPAL* and *Rr4CL*; this finding demonstrates that melatonin can promote the expression levels of these key genes in the phenylpropane synthesis pathway, thereby enhancing flavonoid biosynthesis [38].

The enzymatic generation of flavonols bifurcates into two branches: one, catalyzed by FNS, generates flavonoids, whereas the other, catalyzed by F3H, diverts carbon flow into the synthesis of flavonols and anthocyanins. In this study, the increase in upstream flavonoids in the anthocyanin synthesis pathway and the significant upregulation of terminal gene *RrANS* may contribute to melatonin-induced anthocyanin accumulation. Studies have shown a positive correlation between the expression level of *Rr4CL* and the content of anthocyanins [39]. Moreover, the overexpression of *RrANS* increases anthocyanin accumulation, resulting in a deep red color [40]. These findings indicate that the two are important enzymes in roses that respond to melatonin to promote anthocyanin accumulation. Studies have reported that peonidin 3-O-glucoside may be the main component that determines its red color. This finding suggests that the increase in its content may be a possible reason why rose petals respond to melatonin signals by showing a deep red color. The phenomenon that the contents of original anthocyanin, including procyanidin B1 and B3, was significantly decreased under melatonin treatment, may be due to the competition in the shared upstream common phenylpropane and core flavonoid metabolic pathways, where many upstream substrates flow into anthocyanin synthesis [41].

In this study, silencing *Rr4CL3* reduces petal color, corresponding to a decrease in flavonoid compounds and anthocyanin content; this finding indicates that although *Rr4CL3* is upstream in the flavonoid synthesis pathway, it positively regulates the accumulation of downstream metabolites, such as flavonoid compounds and anthocyanins [42,43]. This outcome possibly results from the upregulation of *Rr4CL3* expression that increases the synthesis substrate naringenin chalcone for flavonoid compounds and anthocyanins, thereby initiating a signaling cascade. After exogenous melatonin treatment, the relative expression level of the *Rr4CL3* gene in silenced petals increases, and the contents of flavonoid compounds and anthocyanins are significantly higher than those in untreated silenced petals. This finding indicates that melatonin can affect the expression levels of functional genes in the synthesis pathway to promote the synthesis of flavonoid compounds and anthocyanins in roses. Moreover, melatonin treatment allows the content of flavonoid compounds in silenced petals to reach levels similar to those in controls, indicating that the *Rr4CL3* gene is one of the key genes in roses responding to melatonin for flavonoid compound synthesis. However, melatonin treatment increases the anthocyanin content in silenced petals to a level that is significantly higher than that in control. This finding indicates that melatonin can also activate *Rr4CL3* gene expression to promote anthocyanin accumulation in roses, even though it is not the key gene that induces anthocyanin increase. Other structural genes or transcription factors serving as key genes that respond to melatonin signals, such as *MYB* transcription factors and *bHLH* transcription factors, may exist [44,45]. In addition, it is reported that melatonin can also regulate the flowering rhythm by protecting floral organs and participating in photoperiod-induced flower transition [46]. Apart from the effects of melatonin significantly increasing the content of flavonoids by regulating the key genes in rose, the impact of melatonin on changing the timing of developmental switches on the synthesis of flavonoids remains open. As an important hormone in mammals and humans, melatonin can influence circadian and circannual rhythms, and regulate mood, body temperature, metabolism, and immunological responses [47,48], showing that the impact of melatonin solution spray would have a great effect on animals (including insects) in contact with the treatment, in particular. Moreover, when preparing a melatonin solution, it generally needs to be dissolved in an organic reagent, which does not align with the current trend toward environmentally friendly green agriculture. With various nanotechnologies being widely used in modern agriculture, the development of melatonin nanomaterials with low melatonin content, to improve melatonin utilization efficiency, is imperative [28]. Anyway, the application of melatonin in agriculture needs to be based on the study of its impact on ecological safety, especially on life activities such as animals, at least if used in the wild.

## 4. Materials and Methods

### 4.1. Plant Materials and Exogenous Melatonin Treatment

The experiment utilized a 2-year-old potted *R. rugosa* ‘Feng hua’ from the rose germplasm resource nursery at Yangzhou University, Jiangsu Province, China (N 32°23′27.64″, E 119°25′10.23″), cultivated in pots with the same soil. Four different melatonin concentrations (0, 0.1, 0.3, and 0.5 mM) were prepared for the foliar application, during which 0 mM melatonin was used in the control group (CK). Melatonin (Sigma-Aldrich, Milwaukee, WI, USA) stock solution was prepared following the instructions suggested by the manufacturers. Briefly, the volume of the needed melatonin solution was determined, and the corresponding amount of the melatonin power was weighed regarding the concentration of the melatonin solution. The melatonin powder was dissolved in a certain volume of anhydrous methanol and then filled to the determined volume with ultrapure water using a volumetric flask. Since the volume fraction of ethanol in all solutions is 0.1%, the volume of anhydrous methanol used was based on the determined volume of the melatonin solution. It should be mentioned that all procedures were shielded from light. Four experimental groups were set with 6 rose plants in each group. Each 2 rose plants were considered as one replicate, and treatments included three replicates. The roses in the four groups were sprayed with four different concentrations of melatonin solution, respectively. Exogenous melatonin treatments were applied at the budding stage of flowers (5 April 2023). Manual watering was used for spraying melatonin solution (600 mL per group) until the rose leaves were thoroughly wetted every 3 days for half a month, when the sky was completely dark at around 20:00. Fully bloomed petal samples were collected on the third day after the last spraying, immediately frozen in liquid nitrogen, and stored in an ultra-low temperature freezer at −80 °C for subsequent experiments.

### 4.2. Measurement of Melatonin Content

Melatonin content was measured using a plant melatonin ELISA kit (Shanghai Qiaodu Biotechnology Co., Ltd., Shanghai, China), following the manufacturer’s instructions. Briefly, 0.1 g of petal samples, ground in liquid nitrogen, were vortex-homogenized with 1.0 mM of phosphate buffer (pH of 7.2; containing 5% methanol) and centrifuged at 10,000× *g* for 10 min to obtain the supernatant. The sample wells on the microplate were each filled with 10 μL of the supernatant and diluted fivefold with 40 μL of the sample dilution solution. Meanwhile, the standard wells received 50 μL of standards at concentrations of 0, 50, 100, 200, 400, and 800 pg/mL, and the blank wells received nothing. Then, each well, including the standard and sample wells, was filled with 100 μL of horseradish peroxidase-labeled detection antibody and incubated at 37 °C for 1 h. After that, the solution in each microplate well was discarded and the wells were dried with absorbent paper. Then, each well was then filled with diluted wash buffer (wash buffer:distillation water = 1:20), shaken to remove the solution, and patted dry with absorbent paper after letting it stand for 1 min. The microplate was washed 5 times to completely clear the products generated by the reaction between melatonin and antibodies. Finally, 50 μL of substrate A and 50 μL of substrate B were added successively to each well, and the microplate was incubated at 37 °C for 15 min in the dark. After 50 μL of termination solution was added to each well, the remaining antigen bound to the solid phase was calculated based on the absorbance value of the solution measured at 450 nm using an MR-96TC microplate reader (Chengk Instruments, Shanghai, China) in 15 min. Melatonin concentration was calculated from the absorbance values using a standard curve.

### 4.3. Measurement of Chlorophyll Content

The chlorophyll content in the leaves was measured using the ethanol–acetone extraction method [49]. The young leaves from each treatment group were randomly selected at around 10:00, washed with ultrapure water, and dried. After being chopped into small pieces of 0.2 mm × 0.2 mm, 0.4 g of samples was weighed and placed into a 25 mL test tube with 12.5 mL of 80% acetone and 12.5 mL of 95% ethanol. The mixture was mixed thoroughly and kept in the dark at room temperature for 24 h. The absorbance values of the solution were measured using a spectrophotometer at wavelengths of 663 and 645 nm. A mixture of 80% acetone and 95% ethanol in equal volumes was used as a blank control. Chlorophyll content (mg/g) was calculated using the following formula: chlorophyll = [(12.72 × *A*_663_ − 2.59 × *A*_645_) + (22.88 × *A*_645_ − 4.63 × *A*_663_)] × *V*/(1000 × *m*). In the formula, *V* is the volume of the solution (mL), and *m* is the quality of the fresh leaves used (g).

### 4.4. Determination of Total Phenol, Total Flavonoids, and Total Anthocyanins

First, 0.2 g of petal samples was ground into powder in liquid nitrogen. Then, they were mixed with 5 mL of 60% anhydrous ethanol and subjected to ultrasonic treatment at 40 °C for 60 min. The mixture was centrifuged at 5000 rpm for 10 min at 4 °C, and the supernatant was collected. This process was repeated twice. Total phenol content was determined using the Folin–Ciocalteu colorimetric method [50]. A standard curve was prepared using a gallic acid standard solution at a concentration of 0.25 mg/mL. The absorbance was measured using a UV–visible spectrophotometer (Mapada-P1, Shanghai, China) at 765 nm. The standard curve equation was Y = 0.0116X + 0.0138 (*R*^2^ = 0.9985). The total phenol content was expressed as mg gallic acid equivalent per 100 g of fresh weight (mg GAE/100 g FW).

The extraction method for total flavonoids was the same as that for the total phenols described above. Total flavonoid content was determined using the NaNO_2_–Al(NO_3_)_3_ method, with rutin being the standard [51]. Absorbance was measured at 510 nm using a UV–visible spectrophotometer. A standard curve with rutin was constructed: Y = 0.0011X + 0.0008 (*R*^2^ = 0.9997). Total flavonoid content was calculated using the standard curve and expressed as mg catechin equivalent per 100 g of fresh weight (mg QE/100 g FW).

For anthocyanins, extraction and determination were performed using cyanidin-3-glucoside (C3G) as the standard, following the methods described by Lee et al. and He et al. [52,53]. Briefly, 0.2 g of petals were extracted with 2 mL of 1% (*v*/*v*) hydrochloric acid/methanol solution at 4 °C in the dark for 24 h. Absorbance was measured at 530 and 657 nm using a UV–visible spectrophotometer. The relative anthocyanin content is expressed as follows: ((*A*_530_ − *A*_657_) × dilution factor/mg FW tissue) × 1000. Total anthocyanin content was calculated based on the calibration curve of C3G and expressed as equivalents of C3G per 1 g fresh weight.

### 4.5. Flavonoids and Anthocyanins Extraction and Metabolomics Analysis

Flavonoid content was analyzed by MetWare (http://www.metware.cn/, accessed on 10 May 2023) using the AB Sciex QTRAP 6500 LC-MS/MS platform. Flower samples were freeze-dried and ground into powder (30 Hz, 1.5 min). Then, 20 mg of the powder was extracted with 0.5 mL of 70% methanol. An internal standard (10 μL of 4000 nmol/L) was added for quantification. The extract was sonicated for 30 min and centrifuged at 12,000 *g* at 4 °C for 5 min. The supernatant was filtered through a 0.22 μm membrane filter. The samples were analyzed using a UPLC-ESI-MS/MS system (UPLC, ExionLC AD, https://sciex.com.cn/, accessed on 10 May 2023; MS, Applied Biosystems 6500 Triple Quadrupole, https://sciex.com.cn/, accessed on 10 May 2023). The UPLC conditions were set as follows: column: Waters ACQUITY UPLC HSS T3 C18 (1.8 µm, 100 mm × 2.1 mm); solvent system: water with 0.05% formic acid (A), acetonitrile with 0.05% formic acid (B); gradient elution program: 0–1 min, 10–20% B; 1–9 min, 20–70% B; 9–12.5 min, 70–95% B; 12.5–13.5 min, 95% B; 13.5–13.6 min, 95–10% B; 13.6–15 min, 10% B; flow rate: 0.35 mL/min; temperature: 40 °C; injection volume: 2 μL. The ESI-MS/MS conditions were set as follows: ion source: ESI+/−; source temperature: 550 °C; ion spray voltage (IS): 5500 V (positive), −4500 V (negative); curtain gas (CUR): 35 psi. Flavonoids were analyzed using scheduled multiple reaction monitoring (MRM). Data acquisition was performed using Analyst 1.6.3 software (Sciex). Multiquant 3.0.3 software (Sciex) was used for quantifying all metabolites. Mass spectrometer parameters, including the declustering potentials and collision energies for individual MRM transitions, were optimized. A specific set of MRM transitions was monitored for each period according to the metabolites eluted during that period.

Anthocyanin content was also detected by MetWare (http://www.metware.cn/, accessed on 10 May 2023) using the AB Sciex QTRAP 6500 LC-MS/MS platform, with slight differences. For the sample preparation and extraction, 50 mg of the powder was extracted with 0.5 mL of methanol/water/hydrochloric acid (500:500:1, *v*/*v*/*v*). The extract was vortexed for 5 min, ultrasonicated for 5 min, and centrifuged at 12,000 *g* at 4 °C for 3 min. The residue was re-extracted by repeating the above steps. The UPLC conditions for anthocyanins were set as follows: column: Waters ACQUITY BEH C18 (1.7 µm, 2.1 mm × 100 mm); solvent system: water with 0.1% formic acid (A), methanol with 0.1% formic acid (B); gradient program: 0 min, 95:5 A/B; 6 min, 50:50 A/B; 12 min, 5:95 A/B (hold for 2 min); 14 min, 95:5 A/B (hold for 2 min). The other conditions were the same as those for flavonoid detection procedures described above.

### 4.6. RNA-seq and Transcriptome Analyses

Transcriptome analysis involving sequencing cDNA libraries was performed using the Illumina platform at Metware Biotechnology Co., Ltd., in Wuhan, China. RNA quality and integrity were assessed using a NanoPhotometer spectrophotometer and an Agilent 2100 bioanalyzer. Library construction utilized the NEBNext Ultra RNA Library Prep Kit, with mRNA enrichment performed using Oligo (dT) magnetic beads. The first cDNA strand was synthesized using the M-MuLV reverse transcriptase system, and the second cDNA strand was generated using the DNA polymerase I system with dNTPs. The RNA library was sequenced on the Illumina HiSeq platform. Raw data were aligned to the reference genome of *R. rugosa* (https://www.rosaceae.org/, accessed on 21 October 2023). Fragments Per Kilobase of transcript per Million fragments mapped (FPKM) were used as an indicator to measure gene expression levels. The threshold for significant differential expression was set at an absolute |log2 Fold Change| ≥ 1.5 and a false discovery rate < 0.05. Gene Ontology (GO) and Kyoto Encyclopedia of Genes and Genomes (KEGG) categories were used to annotate differentially expressed genes (DEGs).

### 4.7. RNA Extraction and qRT-PCR Analysis

RNA was isolated from flower petals using the RNAprep Pure Plant Plus Kit (Polysaccharides and Polyphenolics-rich) (Tiangen, Beijing, China), following the manufacturer’s instructions. Reverse transcription was performed on 2 μg of total RNA to generate first-strand cDNA using the PrimeScript II 1st Strand cDNA Synthesis Kit (Takara, Kyoto, Japan). Then, qRT-PCR was conducted to verify the accuracy of the transcriptome data. The qRT-PCR analysis utilized HiScript III qRT SuperMix and ChamQ SYBR Color qPCR Master Mix (Vazyme, Nanjing, China) on the Bio-Rad CFX96 (Bio-RAD, Hercules, CA, USA). Each sample included three independent biological replicates. Relative gene transcript levels were quantified using the 2^−ΔΔCt^ method based on the Ct values from the CFX manager [54]. The 5.8S rRNA was used as the internal control. The primers are summarized in Supplementary Table S1.

### 4.8. Gene Sequence Analysis and Cloning

Candidate gene *Rr4CL* was identified from the proteome of *R. rugosa* using Geneious 4.8.4 and aligned based on the amino acid sequences using TB tools (https://github.com/CJ-Chen/TBtools, accessed on 5 November 2023). The primers flanking the full-length open reading frames (ORFs) of *Rr4CL* were designed using the NEB Tm Calculator web tool (https://tmcalculator.neb.com/, accessed on 5 November 2023) (Table S1). The ORFs were cloned using 2× KeyPo Master Mix (Dye Plus) (Vazyme, Nanjing, China). PCR products were purified using the FastPure Gel DNA Extraction Mini Kit (Vazyme, Nanjing, China). All PCR products were subcloned into the 5 min TA/Blunt-Zero Cloning vector (Vazyme, Nanjing, China) and transformed into Escherichia coli DH5α competent cells. Positive bacterial clones were confirmed using 2× Rapid Taq Master Mix (Vazyme, Nanjing, China), and PCR products were sequenced at Sangon Biotech (Shanghai, China) using 1% agarose gel electrophoresis.

### 4.9. VIGS

Transient transformations of rose petals (R. chinensis Jacq. ‘Crimson Glory’) using the VIGS method were conducted following the protocols described by Zhang et al. and Wei et al. [39,55]. Fragments specific to *Rr4CL* (300 bp in length) were amplified and inserted into the pTRV2 vector using the ClonExpress II One Step Cloning Kit (Vazyme, Nanjing, China). The primers for the VIGS vectors of the candidate genes are listed in Table S1. The constructed vectors were introduced into the Agrobacterium tumefaciens strain GV3101 and cultured in the LB medium supplemented with 50 mg/mL kanamycin and 25 mg/mL rifampicin on a rocking platform (200 rpm) at 28 °C for 18 h. The Agrobacterium cultures were harvested and resuspended in infiltration buffer (10 mM MgCl_2_, 10 mM 2-morpholinoethanesulphonic acid, 200 μM acetosyringone) to a final OD600 of 1.0. For *Rr4CL*, Agrobacterium cultures containing pTRV1 and either pTRV2-*Rr4CL* or pTRV1 and pTRV2-empty were combined at a 1:1 ratio (*v*/*v*). Fully bloomed rose petals were collected after 3–4 h of incubation in a dark room at room temperature. Two petal discs with a 1 cm diameter were punched out symmetrically from each petal and placed in deionized water to equilibrate. Subsequently, the petal discs were vacuum infiltrated twice (60 s each) in an immersion buffer (−25 kPa). After the vacuum was released, the discs were briefly washed with deionized water, dried, and incubated in the dark at 4 °C for 10 days. The flower disc phenotypes were observed daily and recorded.

### 4.10. Statistical Analysis

Statistical analysis of the data was performed using GraphPad Prism 8.0 (GraphPad Software Inc., San Diego, CA, USA; http://www.graphpad.com/, 5 January 2024). *t*-tests were used for comparisons between two groups (*, *p*-value < 0.05; **, *p*-value < 0.01; ***, *p*-value < 0.001; ****, *p*-value < 0.0001). For multiple groups, a one-way analysis of variance followed by Bonferroni’s post hoc test was performed, with *p*-value < 0.05 considered significant.

## 5. Conclusions

This study focused on the 2-year-old rose cultivar *R. rugosa* ‘Feng hua’ to investigate the effects of exogenous melatonin sprayed on leaves at different concentrations on the biosynthesis of flavonoids in rose petals. Metabolomic and transcriptomic approaches were employed to identify the main components of the secondary metabolites affected by melatonin and the associated differential genes, which were further functionally validated. The results indicate that 0.3 mM exogenous melatonin can improve the content of flavonoid compounds. However, higher concentrations tend to reduce its beneficial effects. Quercitrin, 2″-o-galloylhyperin, and baimaside in flavonols and peonidin-3-o-5-o-(6-o-coumaroyl)-diglucoside and peonidin-3-o-glucoside in anthocyanins are the main factors upregulated by melatonin. A total of 1835 significantly enriched DEGs involved in metabolic pathways, including key structural genes regulating flavonoid and anthocyanin biosynthesis pathways, such as *Rr4CL*, *RrF3H*, and *RrANS*, were identified through transcriptomic analysis. Functional validation confirms that *Rr4CL3* is likely to be a key gene in roses that responds to the melatonin regulation of flavonoid compounds. Furthermore, melatonin can activate *Rr4CL3* gene expression to promote anthocyanin accumulation in rose flowers. In conclusion, this study reveals the regulatory mechanism in which melatonin regulates downstream products in related pathways by modulating structural genes in secondary metabolites, thereby significantly enhancing the flavonoid biosynthesis in *R. rugosa*.

## Data Availability

Data are contained within the article or Supplementary Materials.

## Supplementary Materials

The following supporting information can be downloaded at: https://www.mdpi.com/article/10.3390/ijms25179248/s1.
