# Supplementary material for: Metabolite and Transcriptome Profiling Analysis Provides New Insights into the Distinctive Effects of Exogenous Melatonin on Flavonoids Biosynthesis in Rosa rugosa
Source: Int J Mol Sci. 2024 Aug 26;25(17):9248. doi: 10.3390/ijms25179248 (PMC11395435; doi:10.3390/ijms25179248)
Supplement: Supplementary file 1 [file ijms-25-09248-s001.zip › ijms-3111891-supplementary.pdf]

Table S1. Gene specific primers for qRT-PCR，gene amplification and Virus Induced Gene Silencing (VIGS).

Objective	Gene name	Primer sequences
qRT-PCR	*RrPPDC*	Forward: GCAACTTGGCCGGGATTCCT
	RrPPDC	Reverse: TGCCTCCACTGCAGCCTCTA
	RrAADC	Forward: ACGCCGAATTGGTAGCGAGA
	RrAADC	Reverse: GGCCCGGAAGTTCTCCATGT
	Rr4CL	Forward: GTCGCTCCCTCGGCTACAAC
	Rr4CL	Reverse: CGTGGCCTCATCGTCGTTCA
	RrF3H	Forward: ACGAGAGTGTGGAGCACAGAGT
	RrF3H	Reverse: GCTCCTCCAAGGGCTTCACC
	RrDFR	Forward: AAAGCGGCCGCCGATAAGAT
	RrDFR	Reverse: CATTGCCGGCGGTGAGTTTC
	RrANS	Forward: GGCCTGCAGCTCTTCTACGG
	RrANS	Reverse: TCCAAGGTGTCGCCGATGTG
	5.8s	Forward: CGGCAACGGATATCTCGG
	5.8s	Reverse: TGTGACGCCCAGGCAGACG
Gene amplification	*Rr4CL*	Forward: ATGATATCCATTGCGTCTAACAACAAC
	Rr4CL	Reverse: TTAAACGTTCGGAGTGGCTAGC
VIGS	*TRV2r4CL*	Forward: TGTCTTCGGGACATGCCCGGGTTCAAGTGGTCACCATCGAC
	TRV2r4CL	Reverse: AGAAGGCCTCCATGGGGATCCACAACAAGACACTGTTCAAGGAGT

Table S2 The ratio of mapped reads of individual sequencing fragments compared with the reference genome

Sample	Total Reads	Reads mapped	Unique mapped
CK1	54700118	48663579(88.96%)	45636531(83.43%)
CK2	49891624	44405458(89.00%)	41880392(83.94%)
CK3	53442040	48350097(90.47%)	45540509(85.21%)
MT1	53397962	48349997(90.55%)	45491812(85.19%)
MT2	52600754	47348928(90.02%)	44540578(84.68%)
MT3	57420864	51191748(89.15%)	48155256(83.86%)

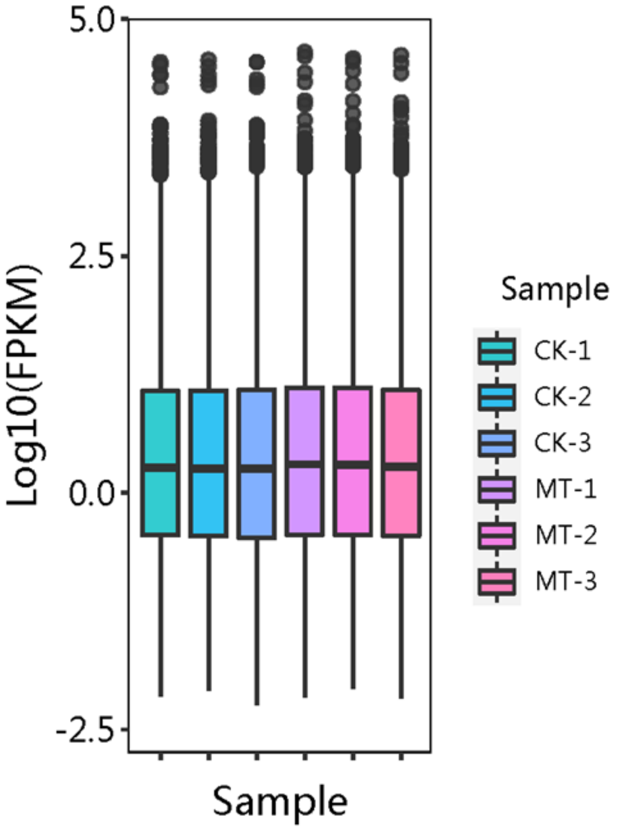

Figure S1. The distribution of FPKM values depicted in the box plot across samples.

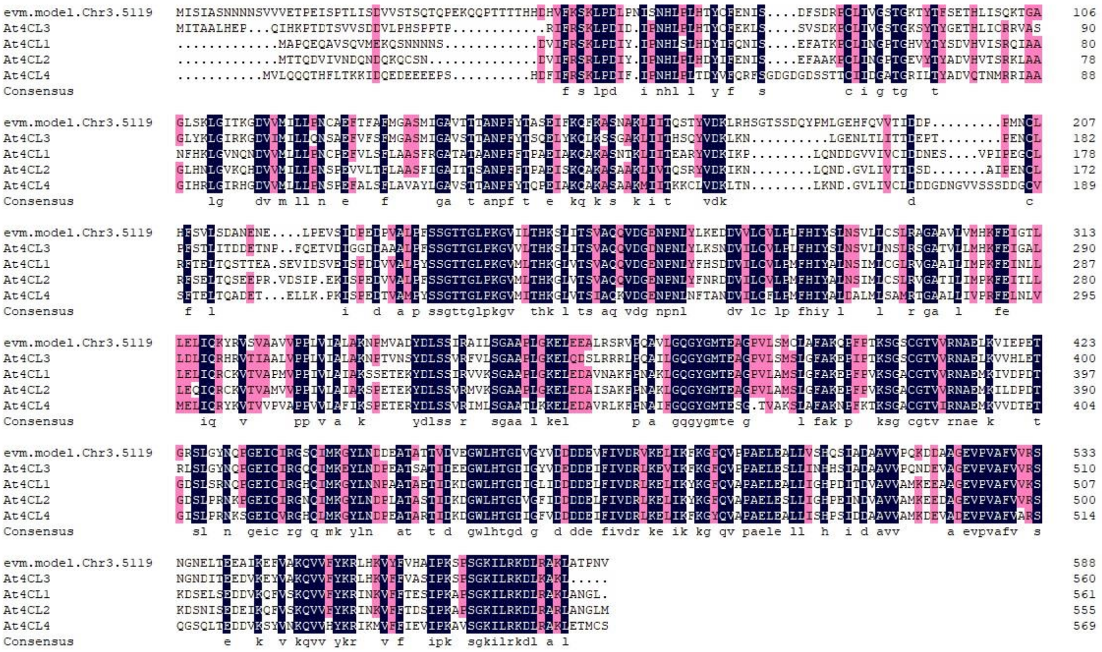

Figure S2. Comparison of Rose *Rr4CL* between four homologous gene sequences of Arabidopsis *At4CL*
